# Development and validation of a nomogram for predicting the efficacy of vidian neurectomy in the treatment of chronic rhinosinusitis with nasal polyps combined with allergic rhinitis

**DOI:** 10.3389/fsurg.2025.1682674

**Published:** 2025-11-18

**Authors:** Cheng-Li Xu, Qiao Huang, Zi-Ang Zhu

**Affiliations:** Department of Otolaryngology Head and Neck Surgery, The Second Affiliated Hospital of Guangxi Medical University, Nanning, China

**Keywords:** vidian neurectomy, chronic rhinosinusitis with nasal polyps and allergic rhinitis, XGBoost model, predictive model, nomogram

## Abstract

**Background:**

Vidian neurectomy (VN) is commonly used to treat chronic rhinosinusitis with nasal polyps combined with allergic rhinitis (CRSwNP with AR). However, its therapeutic efficacy varies among individuals. This study aimed to develop a nomogram to predict treatment efficacy and provide reference for clinical decision-making.

**Methods:**

A total of 350 patients with CRSwNP and AR who underwent VN were retrospectively enrolled and divided into effective and ineffective groups based on treatment outcomes. Univariate analysis was performed to compare demographic and disease-related characteristics between the two groups. Significant variables from the univariate analysis were included as predictors in an XGBoost model, with SHAP visualization used to identify important features. In parallel, multivariate logistic regression was conducted to determine independent predictors of efficacy. Variables identified as both important and statistically significant from these two methods were used to construct a nomogram. The performance of the nomogram was evaluated using calibration curves, receiver operating characteristic (ROC) curves, and decision curve analysis (DCA).

**Results:**

The effective group accounted for 74.57% of the cohort. The ineffective group showed significantly higher values in several indicators, including disease duration, history of endoscopic sinus surgery, inflammatory markers, and symptom scores. Both the XGBoost model and multivariate logistic regression identified preoperative white blood cell count (WBC), operation duration, history of endoscopic sinus surgery, total IgE level, and SNOT-22 score as significant predictors (all *P* < 0.05). The constructed nomogram based on these factors demonstrated good predictive performance (training set AUC = 0.738, validation set AUC = 0.853) and clinical applicability (DCA showed notable net benefit).

**Conclusion:**

This study successfully developed and validated a nomogram incorporating preoperative WBC, operation duration, prior surgical history, total IgE, and SNOT-22 score to predict the efficacy of VN in treating patients with CRSwNP and AR. The model offers a reliable tool to assist clinicians in making personalized treatment decisions.

## 1 Introduction

Chronic sinusitis with nasal polyps (CRSwNP) is a common chronic inflammatory disease of the upper respiratory tract, with a global prevalence of approximately 2%–4% (1) (2),. The disease is mainly characterized by persistent inflammation of the nasal and sinus mucosa and the formation of polyps, often accompanied by allergic rhinitis (AR), with a comorbidity rate of 30%–60% (3, 4). These patients present clinically with stubborn nasal congestion, runny nose, decreased sense of smell, and other symptoms, which seriously affect their quality of life and work efficiency (5, 6).

For patients with moderate to severe CRSwNP complicated with AR, surgical intervention becomes an important treatment option after drug therapy failure (7). Among them, vidian neurectomy (VN) is an important surgical intervention, and its treatment mechanism is mainly based on precise blockade of the parasympathetic nervous pathway (8). This surgery can achieve dual therapeutic effects: on the one hand, it can inhibit excessive secretion of nasal mucosal glands, significantly improving symptoms such as nasal leakage and postnasal drip in patients; On the other hand, it can effectively reduce abnormal dilation of nasal blood vessels and alleviate nasal congestion caused by congestion and edema of the nasal mucosa (9). Clinical studies have shown that this procedure can produce long-lasting efficacy in 80%-90% of patients with indications, especially for moderate to severe patients who have failed conservative drug treatment. However, attention should be paid to possible complications related to nerve transection such as dry eye syndrome (10). With the advancement of nasal endoscopic technology, modern VN surgery has achieved more accurate nerve localization and smaller tissue trauma, becoming one of the important functional surgical choices in the field of rhinology. However, clinical practice has shown that there are significant individual differences in the efficacy of VN, and some patients do not show significant improvement in postoperative symptoms, which poses a huge challenge to clinical decision-making.

To further improve the accuracy of the predictive model, this study employed the XGBoost (eXtreme Gradient Boosting) model, which is an ensemble learning method based on gradient boosting trees (Gradient Boosting Tree) and can effectively handle nonlinear relationships and complex interactions among features (11). In addition, SHAP (SHapley Additive exPlanations) values were used to quantify and visualize the contribution of each feature to the prediction. Currently, XGBoost models with SHAP-based visualization have been widely applied in the development of medical predictive models and clinical decision support, for example, predicting the risk of breast cancer recurrence and sarcopenia in postoperative gastric cancer patients (12, 13).

At present, research on VN mainly focuses on comparing its efficacy with other surgical treatment methods, and research on predicting the efficacy of VN is relatively scarce (14). This study systematically analyzed the potential factors that affect the efficacy of VN, including demographic characteristics, clinical indicators, laboratory tests, and imaging parameters. Using XGBoost algorithm for feature importance ranking, combined with multiple factor logistic regression analysis to determine independent predictive factors, and finally constructing and verifying a column chart prediction model. We hope to provide objective and quantitative decision-making tools for clinical doctors, achieve truly personalized and precise treatment, and improve patient prognosis.

## 2 Materials and methods

### 2.1 Study population

This retrospective study included 350 patients with chronic rhinosinusitis with nasal polyps combined with allergic rhinitis (CRSwNP with AR) who underwent vidian neurectomy (VN) at our hospital from July 2018 to July 2024. All patients met the diagnostic criteria for CRSwNP as outlined in the “Chinese Guidelines for the Diagnosis and Treatment of Chronic Rhinosinusitis (2018 edition)” and the diagnostic criteria for AR as per the “Allergic Rhinitis and its Impact on Asthma (ARIA) guidelines-2016 revision” (15). Inclusion criteria were: (1) age ≥18 years; (2) confirmed diagnosis of CRSwNP with AR, and ineffective standardized drug treatment for more than 6 weeks; (3) received unilateral or bilateral VN treatment. Exclusion criteria were: (1) presence of other nasal diseases (e.g., nasal tumors, fungal sinusitis); (2) severe hepatic or renal dysfunction; (3) severe systemic diseases or immune system disorders such as malignancies; (4) severe psychiatric disorders or poor compliance; (5) missing key data.

### 2.2 Surgical procedure

The patient was placed in the supine position, and routine disinfection and draping were performed. Both nasal cavities were treated with cotton pledgets containing a small amount of epinephrine to induce mucosal vasoconstriction. Endoscopic vidian neurectomy, pterygopalatine (sphenopalatine) nerve transection, and anterior ethmoidal nerve transection were then performed sequentially: the sphenopalatine foramen and related nerve plexuses were exposed, the surrounding mucosa was circumferentially incised, and the vidian nerve and its pharyngeal branches were transected, with hemostasis achieved by electrocautery. After bilateral nerve transection, functional endoscopic sinus surgery (FESS) was performed, including removal of middle meatus polyps and opening of the natural ostia of the ethmoid, sphenoid, frontal, and maxillary sinuses, preserving necessary drainage pathways and trimming mucosal edges to ensure sinus patency. The procedure was completed successfully, and the patient was returned to the ward.

### 2.3 Data collection

The indicators collected in this study included: age, gender, body mass index (BMI), smoking status, disease duration, presence of asthma, history of prior nasal endoscopic surgery, family history of allergies, preoperative sinonasal outcome test-22 (SNOT-22) score (0–110), preoperative total nasal symptom score (TNSS) score (0–12), nasal congestion VAS score (0–10), rhinorrhea VAS score (0–10), sneezing VAS score (0–10), preoperative Lund-Kennedy endoscopic score (0–12) (16), type of surgery, surgical laterality, operation duration, intraoperative blood loss, intraoperative complications, postoperative bleeding severity, length of hospital stay, postoperative dryness, postoperative crusting, postoperative facial numbness, preoperative C-reactive protein (CRP), preoperative eosinophil count, preoperative total Immunoglobulin E (IgE), preoperative basophil count, and total white blood cell count (WBC). According to previous literature, patients were classified into the effective group if their postoperative total SNOT-22 score decreased by ≥30% compared to preoperative scores and the Lund-Kennedy endoscopic score improved significantly (score decrease ≥1 point); otherwise, they were classified into the ineffective group. This classification indicated whether patients achieved significant improvement in both symptoms and endoscopic findings.

### 2.4 Statistical analysis

All analyses and plotting in this study were performed using R software version 4.4.1. Continuous variables were expressed as medians (minimum–maximum) and compared between groups using *t*-test or Mann–Whitney *U*-test depending on distribution characteristics. Categorical variables were presented as counts (percentages) and compared using Chi-square test or Fisher's exact test. The dataset was split into training and validation sets at a ratio of 7:3. Significant factors from univariate analysis were used as independent variables, with treatment efficacy (effective vs. ineffective) as the dependent variable. In the training set, XGBoost model parameters were tuned using the train function with 10-fold cross-validation and grid search to optimize multiple hyperparameters. After obtaining the best parameter combination, the final model was constructed using the xgboost function based on optimal parameters. SHAP values were calculated using the shapviz package to interpret the positive or negative contribution of each variable to the prediction outcome, and feature importance plots were drawn. In the validation set, confusion matrix was created and model performance was evaluated by F1 score, accuracy, recall, precision, and ROC curve. To further assess the robustness and generalizability of the model, RF, SVM, and MLP models were constructed using the same training and validation sets, and their performance was evaluated using the same metrics. Using the same independent and dependent variables, multivariate logistic regression analysis was conducted to identify independent factors associated with treatment efficacy. Variables from the feature importance plot were sequentially verified for significance in the multivariate logistic regression, and those significant were selected until five variables were chosen. Based on these five important and significant factors, a nomogram was constructed in both training and test sets. The model's predictive performance was assessed by ROC curve, calibration curve to evaluate consistency between predicted and observed outcomes, and decision curve analysis (DCA) to evaluate clinical utility.

## 3 Results

### 3.1 Comparison of baseline characteristics and surgery-related information between the effective and ineffective groups of patients with chronic rhinosinusitis with nasal polyps and allergic rhinitis

A total of 350 patients with chronic rhinosinusitis with nasal polyps combined with allergic rhinitis were included, of whom 261 cases (74.57%) were evaluated as “effective” after surgery, and 89 cases (25.43%) as “ineffective.” The disease duration in the ineffective group was significantly longer than in the effective group (*P* = 0.0258), and the proportion of patients with a history of prior nasal endoscopic surgery was higher (*P* < 0.0001). Preoperative SNOT-22 scores were significantly higher in the ineffective group compared to the effective group (*P* = 0.0202), as were preoperative TNSS scores (*P* = 0.026), nasal congestion VAS scores (*P* = 0.00699), sneezing VAS scores (*P* = 0.017), and Lund-Kennedy scores (*P* = 0.00504) (Table 1). The ineffective group also had longer operation times (*P* = 0.000183), more severe postoperative bleeding (*P* < 0.0001), higher incidence of postoperative dryness (*P* = 0.0292), significantly higher peripheral blood eosinophil counts (*P* = 0.0414), higher total IgE levels (*P* = 0.00437), and higher white blood cell counts (*P* = 0.00118) than the effective group (Table 2). A total of 3 cases (0.86%) of intraoperative complications were observed, all presenting as mild and reversible palatal numbness. Postoperative bleeding occurred during hospitalization, mainly as mild oozing, which was well controlled with routine management. No severe bleeding or reoperation was required.

**Table 1:** Baseline characteristics between effective and ineffective groups.

Variables	All Patients (*n* = 350)	Effective (*n* = 261)	Ineffective (*n* = 89)	*P*-value
Age	47 (27–63)	47 (27–63)	47 (28–63)	0.327
Gender				0.3968668
Male	189 (54%)	137 (52.49%)	52 (58.43%)	
Female	161 (46%)	124 (47.51%)	37 (41.57%)	
BMI	23.7 (18.6–29.2)	23.8 (18.6–29.2)	23.7 (18.7–29.1)	0.908
Smoking				0.1240324
No or Mild	301 (86%)	226 (86.59%)	75 (84.27%)	
Moderate	32 (9.14%)	20 (7.66%)	12 (13.48%)	
Severe	17 (4.86%)	15 (5.75%)	2 (2.25%)	
Disease duration	9.0 (3.5–14.8)	8.6 (3.5–14.8)	10.3 (3.6–14.8)	0.0258
Comorbid asthma				0.5174226
Yes	76 (21.71%)	54 (20.69%)	22 (24.72%)	
No	274 (78.29%)	207 (79.31%)	67 (75.28%)	
History of prior nasal endoscopic surgery				6.42E-06
Yes	136 (38.86%)	83 (31.8%)	53 (59.55%)	
No	214 (61.14%)	178 (68.2%)	36 (40.45%)	
Family history of allergy				0.1328559
Yes	132 (37.71%)	92 (35.25%)	40 (44.94%)	
No	218 (62.29%)	169 (64.75%)	49 (55.06%)	
Sinonasal Outcome Test-22 (SNOT-22)	48 (38–59)	48 (38–59)	50 (38–59)	0.0202
Total Nasal Symptom Score (TNSS)	9 (6–12)	9 (6–12)	10 (6–12)	0.026
Nasal congestion VAS score	6 (4–8)	6 (4–8)	7 (4–8)	0.139
Rhinorrhea VAS score	5 (3–8)	5 (3–8)	6 (3–8)	0.00699
Sneezing VAS score	5 (3–7)	5 (3–7)	6 (3–7)	0.017
Lund-Kennedy Endoscopic Scoring System (Lund-Kennedy)	8 (5–11)	8 (5–11)	9 (5–11)	0.00504

**Table 2:** Surgical characteristics between effective and ineffective groups.

Variables	All Patients (*n* = 350)	Effective (*n* = 261)	Ineffective (*n* = 89)	*P*-value
Type of surgery				0.6134105
Isolated pterygopalatine nerve transection	124 (35.43%)	90 (34.48%)	34 (38.2%)	
Combined surgery involving pterygopalatine nerve transection	226 (64.57%)	171 (65.52%)	55 (61.8%)	
Surgical side				0.4374941
unilateral	132 (37.71%)	102 (39.08%)	30 (33.71%)	
bilateral	218 (62.29%)	159 (60.92%)	59 (66.29%)	
Duration of surgery (minutes)	91 (45–135)	82 (45–135)	103 (45–134)	0.000183
Intraoperative blood loss (mL)	38 (14–60)	38 (14–60)	38 (14–60)	0.743
Intraoperative complications				0.3263208
Yes	3 (0.86%)	1 (0.38%)	2 (2.25%)	
No	347 (99.14%)	260 (99.62%)	87 (97.75%)	
Postoperative bleeding				1.50E-05
Mild	320 (91.43%)	249 (95.4%)	71 (79.78%)	
Moderate	30 (8.57%)	12 (4.6%)	18 (20.22%)	
Length of hospital stay (days)	5 (3–7)	5 (3–7)	5 (3–7)	0.603
Postoperative dryness				0.02918381
Yes	156 (44.57%)	107 (41%)	49 (55.06%)	
No	194 (55.43%)	154 (59%)	40 (44.94%)	
Postoperative crusting				0.197288
Yes	120 (34.29%)	84 (32.18%)	36 (40.45%)	
No	230 (65.71%)	177 (67.82%)	53 (59.55%)	
Postoperative facial numbness				0.06471045
Yes	112 (32%)	76 (29.12%)	36 (40.45%)	
No	238 (68%)	185 (70.88%)	53 (59.55%)	
C-reactive protein (CRP) (mg/L)	4.19 (2.23–6.48)	4.19 (2.23–6.48)	4.19 (2.26–6.45)	0.571
Eosinophil count (×10^9^/L)	0.83 (0.32–1.43)	0.81 (0.32–1.43)	0.91 (0.32–1.43)	0.0414
Total Immunoglobulin E (IU/mL)	535.10 (197.37–850.57)	505.17 (198.13–850.57)	600.19 (197.37–841.55)	0.00437
Basophils count (×10^9^/L)	0.08 (0.02–0.13)	0.07 (0.02–0.13)	0.08 (0.02–0.13)	0.164
White Blood Cell count (×10^9^/L)	9.02 (6.87–11.34)	8.86 (6.87–11.34)	9.60 (6.97–11.29)	0.00118

### 3.2 Important features selected by the XGBoost model

**Figure 1:**
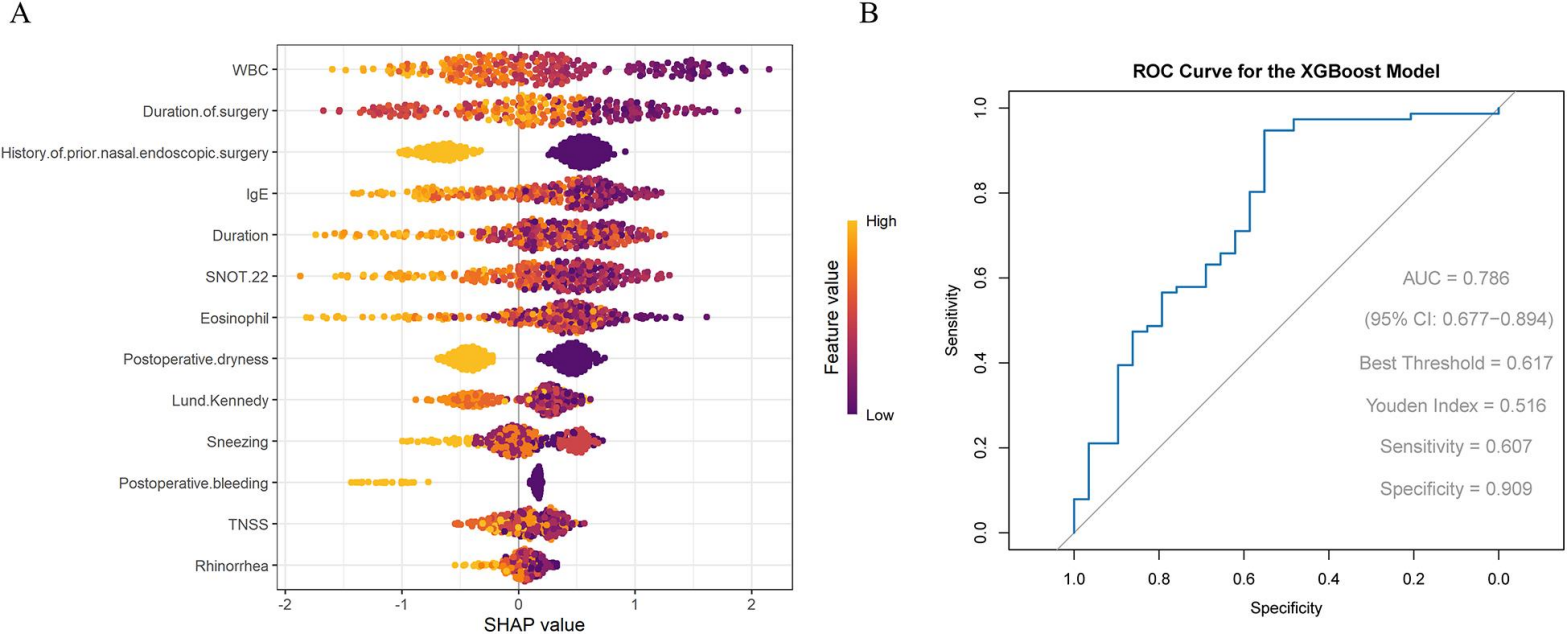
**(A)** SHAP visualization results of the XGBoost model **(B)** ROC curve evaluating the performance of the XGBoost model.

Significant factors from the univariate analysis above were further screened using the XGBoost model. The results indicated that WBC, operation duration, history of prior nasal endoscopic surgery, total IgE level, and disease duration were the most contributory factors (Figure 1A). The ROC curve showed that the XGBoost model had an AUC of 0.786, with sensitivity and specificity of 0.607 and 0.909, respectively. The F1 score was 87.12%, accuracy 80%, recall 92.21%, and precision 82.56%. a positive predictive value (PPV) of 82.35%, and a negative predictive value (NPV) of 70%. These results indicate that the model performed particularly well in recall and precision, effectively identifying most patients with effective treatment while maintaining a low false-positive rate (Figure 1B).

To further validate model performance, the XGBoost model was compared with RF, SVM, and MLP models. The results showed that, except for recall, the performance metrics of RF and SVM were generally lower than those of XGBoost; the performance metrics of MLP were lower than those of XGBoost across all measures (Supplementary Table S1; Supplementary Figure S1A–C). Overall, XGBoost demonstrated the most balanced and superior performance in terms of AUC, accuracy, and precision, and was therefore selected as the optimal predictive model in this study.

### 3.3 Multivariate logistic regression analysis identifying independent influencing factors

The results showed that previous nasal endoscopic surgery history (*P* < 0.001, OR = 0.820, 95% CI: 0.754–0.892), SNOT-22 score (*P* = 0.041, OR = 0.993, 95% CI: 0.986–1.000), sneezing score (*P* = 0.001, OR = 0.948, 95% CI: 0.918–0.979), operative time (*P* < 0.001, OR = 0.997, 95% CI: 0.996–0.999), total white blood cell count (WBC) (*P* = 0.001, OR = 0.948, 95% CI: 0.918–0.979), postoperative dryness (*P* = 0.012, OR = 0.900, 95% CI: 0.829–0.976), and postoperative bleeding (*P* < 0.001, OR = 0.748, 95% CI: 0.647–0.865) were significant factors affecting treatment efficacy, all showing negative correlations. Other factors such as disease duration, nasal obstruction, rhinorrhea, eosinophil count, and Lund-Kennedy score did not show statistical significance (*P* > 0.05). The model intercept was significant (*P* < 0.001), indicating a good model fit (Table 3).

**Table 3:** Multivariate logistic regression analysis identified significant factors influencing efficacy.

Term	Estimate	Std error	Statistic	*P* value	OR	CI-lower	CI-upper
Duration	−0.009	0.006	−1.458	0.146	0.991	0.979	1.003
History of prior nasal endoscopic surgery	−0.198	0.043	−4.624	0.000	0.820	0.754	0.892
SNOT 22	−0.007	0.003	−2.054	0.041	0.993	0.986	1.000
TNSS	−0.009	0.012	−0.798	0.426	0.991	0.968	1.014
Rhinorrhea	−0.018	0.014	−1.299	0.195	0.982	0.957	1.009
Sneezing	−0.054	0.017	−3.251	0.001	0.948	0.918	0.979
Duration of surgery	−0.003	0.001	−3.768	0.000	0.997	0.996	0.999
Eosinophil	−0.104	0.063	−1.661	0.098	0.901	0.796	1.019
IgE	0.000	0.000	−2.714	0.007	1.000	1.000	1.000
WBC	−0.054	0.016	−3.290	0.001	0.948	0.918	0.979
Postoperative dryness	−0.106	0.042	−2.539	0.012	0.900	0.829	0.976
Postoperative bleeding	−0.290	0.074	−3.909	0.000	0.748	0.647	0.865
Lund Kennedy	−0.021	0.011	−1.803	0.072	0.979	0.958	1.002

### 3.4 Construction and validation of the nomogram

**Figure 2:**
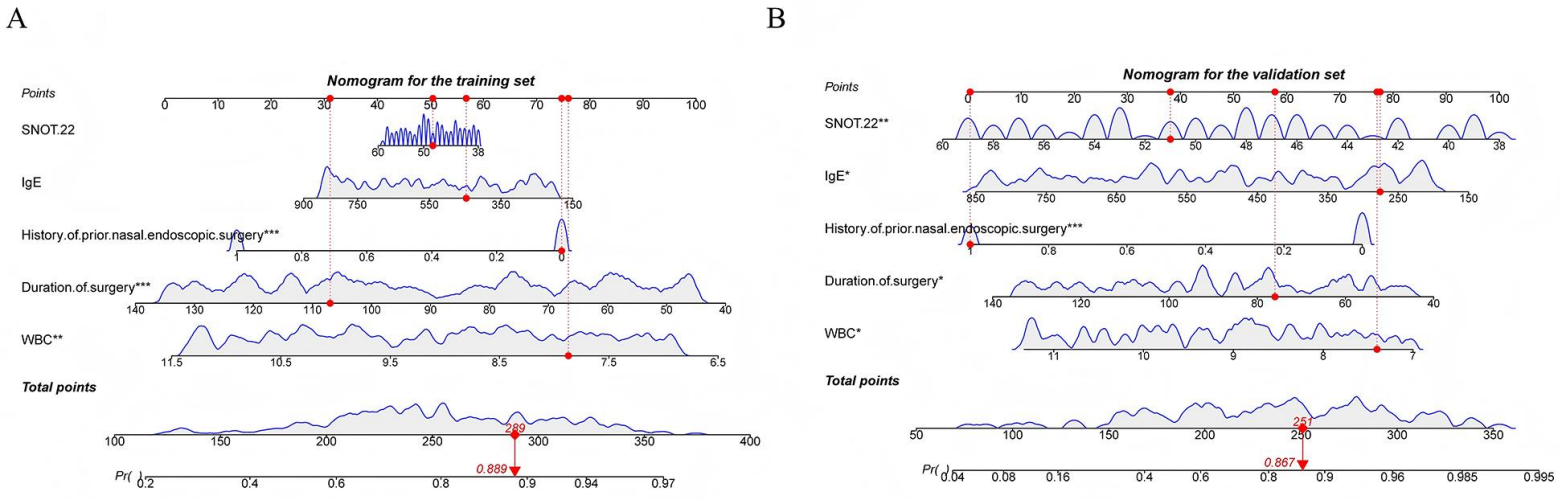
**(A)** Nomogram constructed in the training set **(B)** nomogram constructed in the validation set.

**Figure 3:**
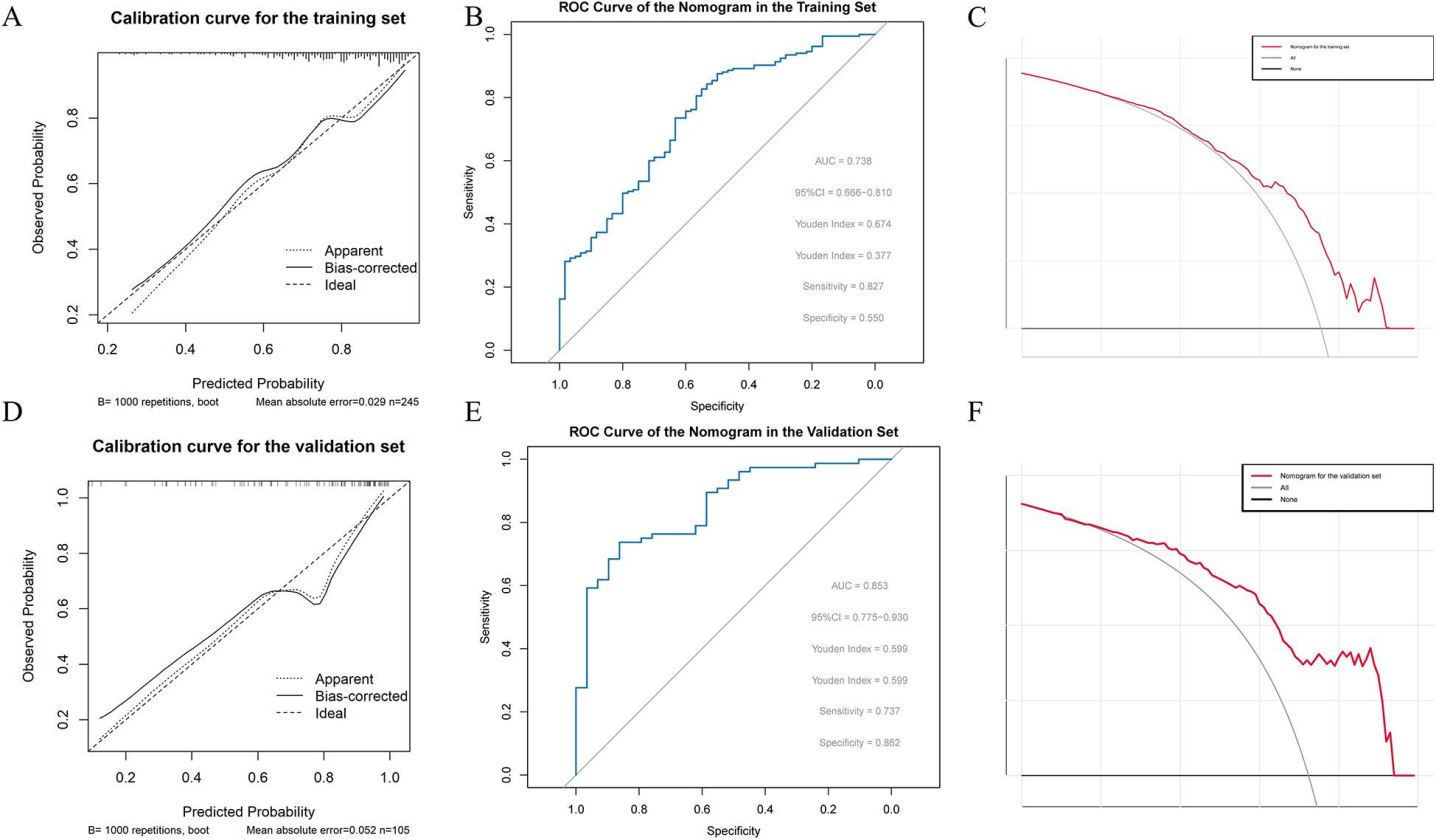
**(A)** Calibration curve of the nomogram in the training set **(B)** ROC curve of the nomogram in the training set **(C)** decision curve analysis (DCA) of the nomogram in the training set **(D)** calibration curve of the nomogram in the validation set **(E)** ROC curve of the nomogram in the validation set **(F)** decision curve analysis (DCA) of the nomogram in the validation set.

Following the steps described in the methods, five important and significant factors were finally selected: preoperative WBC level, operative time, previous nasal endoscopic surgery history, preoperative total IgE level, and preoperative SNOT-22 score. A nomogram was constructed based on these factors. Each factor has a corresponding scale, and patients receive points according to their individual conditions. The total points summed across these five factors correspond to a “predicted probability” of treatment efficacy on the bottom axis. For example, a patient in the training set with a total score of 289 corresponds to an 88.9% probability of effective treatment, while a patient in the validation set with a total score of 251 corresponds to an 86.7% predicted probability of treatment efficacy (Figures 2A,B). The nomogram's performance was evaluated separately in the training and validation sets. Calibration curves showed good agreement between predicted and observed probabilities, with the curves closely following the ideal diagonal line, indicating good calibration ability. ROC analysis yielded AUC values of 0.738 and 0.853, demonstrating good discriminative ability of the nomogram in differentiating outcomes. Decision curve analysis (DCA) showed that the nomogram's net benefit was significantly higher than that of the two extreme strategies of “treat all” and “treat none” (Figures 3A–F).

## 4 Discussion

This study demonstrates that preoperative WBC level, surgery duration, history of previous nasal endoscopic surgery, preoperative total IgE level, and preoperative SNOT-22 score are factors influencing the efficacy of vidian neurectomy (VN) in treating chronic rhinosinusitis with nasal polyps combined with allergic rhinitis. WBC is commonly used as an indicator of systemic or local inflammatory response to evaluate the body's immune status and infection condition. A higher preoperative WBC level indicates a more severe systemic inflammatory state, which can lead to irreversible structural changes such as nasal polyp fibrosis and epithelial damage, as well as mucosal edema and increased purulent secretions (17). Even after surgery, these established lesions are difficult to fully reverse. Elevated WBC may also imply increased levels of cytokines like IL-4, IL-5, and IL-13, resulting in more severe nasal obstruction, rhinorrhea, and loss of smell (18). These symptoms are not only driven by cholinergic nerves but also regulated by eosinophils, IgE, and IL-5 (19). Since VN only reduces parasympathetic nerve influence and cannot directly suppress these pathways, patients respond poorly to neurectomy (20). Longer surgery duration is associated with poorer outcomes, possibly because a longer operation reflects more severe disease, wider polyp involvement, and more intense mucosal inflammation. VN cannot fully reverse established structural changes (such as polyps and scars), limiting postoperative improvements in Lund-Kennedy scores. Prolonged mechanical stimulation during surgery may worsen mucosal injury and promote the release of pro-inflammatory mediators, increasing postoperative inflammation, mucosal edema, and secretions, thus raising Lund-Kennedy scores. A wider nasal polyp area can also cause poor surgical field visibility, affecting precise nerve transection and potentially leading to increased bleeding and delayed tissue repair, thereby reducing efficacy. Patients with a history of prior nasal endoscopic surgery show poorer outcomes, possibly because previous surgery causes middle turbinate defects or sinus adhesions, making it difficult to completely transect the pterygopalatine ganglion during VN. Scar tissue formed previously is more prone to bleeding, which reduces the clarity of the surgical field and causes incomplete nerve transection, lowering treatment efficacy. Higher preoperative total IgE levels also predict poorer outcomes. Total IgE, secreted by plasma cells into blood and tissue, is closely related to allergic reactions, and its elevation worsens allergic symptoms (21, 22). For CRSwNP patients with AR in this study, increased total IgE usually indicates the presence of a Th2-type inflammation, which promotes mast cells or basophils to release histamine, leukotrienes, IL-4, and IL-13, causing nasal mucosal edema, itching, and increased secretions (23). VN only blocks parasympathetic nerves and cannot inhibit these physiological processes, so postoperative Lund-Kennedy scores and quality of life improvements are limited. Preoperative SNOT-22 scores negatively correlate with treatment efficacy through similar mechanisms. Higher SNOT-22 scores reflect a larger nasal polyp burden, more severe nasal mucosal inflammation, and irreversible structural changes that limit the effect of VN. Moreover, SNOT-22 includes systemic symptoms such as sleep disturbances, decreased concentration, and mood decline (24, 25). Higher scores indicate more severe systemic symptoms, which are also less likely to improve given the limited mechanism of VN.

This study integrates results from both the XGBoost model and multivariate logistic regression to construct a nomogram predicting the efficacy of VN. The scoring system quantifies each factor's contribution to treatment outcome and generates individualized probabilities, visually highlighting variable importance and facilitating comparison of the influence strength among factors. This clarifies core intervention targets. The nomogram also helps identify high-risk patients with predicted poor efficacy early, guiding clinicians to implement intensified intervention strategies (e.g., combined biologic therapy) and schedule closer endoscopic follow-up.

Although endoscopic sinus surgery (ESS) alone can effectively improve nasal obstruction, rhinorrhea, and olfactory impairment in patients with CRSwNP combined with AR, several studies have shown that additional Vidian neurectomy (VN) can provide more significant relief of nasal reflex symptoms and yield greater improvements in the Lund-Kennedy score as well as in allergy-related quality-of-life assessments such as SNOT-22 and TNSS. Moreover, current evidence indicates that the combination of ESS and VN is generally safe and does not increase the risk of complications (e.g., dry eye), nor does it adversely affect patients' long-term quality of life. Therefore, ESS combined with VN offers an integrated surgical strategy that balances efficacy and safety for patients with CRSwNP and AR (8, 26).

There are limitations in this study. First, as a retrospective study, data selection bias may exist. Second, it is a single-center study with a relatively small sample size. Confounding factors such as IL-4 and IL-5 were not fully excluded. Although a predictive model for VN efficacy was established, biological mechanisms discussed remain insufficiently explored. Future research should involve multicenter prospective randomized controlled trials to validate these findings. In this study, the ROC curve was not sufficiently smooth, which may be due to the relatively small sample size leading to limited calculation points for sensitivity and specificity, resulting in a less smooth curve. Although this does not affect the interpretation of the AUC, future studies with larger sample sizes are warranted to further validate the stability and generalizability of the model.

## 5 Conclusion

This study identified factors influencing the efficacy of VN for chronic rhinosinusitis with nasal polyps combined with allergic rhinitis using the XGBoost model and multivariate logistic regression analysis. These factors include preoperative WBC level, surgery duration, history of previous nasal endoscopic surgery, preoperative total IgE level, and preoperative SNOT-22 score. A nomogram based on these factors demonstrated good predictive performance. However, it should be noted that surgical duration cannot be determined preoperatively, so the practical applicability of this nomogram may be relatively limited. Nevertheless, the results of this study provide a certain reference for predicting the efficacy of ESS combined with VN.

## Data Availability

The original contributions presented in the study are included in the article/Supplementary Material, further inquiries can be directed to the corresponding author.

## Supplementary material

Supplementary Figure S1 **(A)** ROC curve for RF model **(B)** ROC curve for SVM model **(C)** ROC curve for MLP model.

## References

[1] Keating MK Phillips JC Phillips J. Chronic rhinosinusitis. Am Fam Physician. (2023) 108(4):370–7. 37843944

[2] Kwah JH Peters AT. Nasal polyps and rhinosinusitis. Allergy Asthma Proc. (2019) 40(6):380–4. 10.2500/aap.2019.40.4252 31690375

[3] Avdeeva K Fokkens W. Precision medicine in chronic rhinosinusitis with nasal polyps. Curr Allergy Asthma Rep. (2018) 18:4–25. 10.1007/s11882-018-0776-8 29574586 PMC5866836

[4] Slavin RG. Nasal polyps and sinusitis. JAMA. (1997) 278(22):1849–54. 10.1001/jama.1997.03550220055009 9396646

[5] Corey JP. Chronic rhinosinusitis with nasal polyps (CRSwNPs) and allergic rhinitis (AR). Am J Rhinol Allergy. (2013) 27(6):441–3. 10.2500/ajra.2013.27.0016 24274216

[6] Ren F Zhang L Zhao D Zhang J. Association between allergic rhinitis, nasal polyps, chronic sinusitis and chronic respiratory diseases: a Mendelian randomization study. BMC Pulm Med. (2025) 25:1–109. 10.1186/s12890-025-03523-1 40069797 PMC11899691

[7] Eschenbacher W Straesser M Knoeddler A Li RC Borish L. Biologics for the treatment of allergic rhinitis, chronic rhinosinusitis, and nasal polyposis. Immunol Allergy Clin North Am. (2020) 40(4):539–47. 10.1016/j.iac.2020.06.001 33012318 PMC7539135

[8] Kim JS Stybayeva G Hwang SH. Effectiveness of vidian neurectomy in chronic rhinosinusitis with nasal polyps: a systematic review and meta-analysis. Otolaryngol Head Neck Surg. (2025) 172(3):787–97. 10.1002/ohn.1037 39467055

[9] Kim JS Stybayeva G Hwang SH. Efficacy of vidian neurectomy in treating chronic rhinosinusitis with nasal polyps combined with allergic rhinitis: a systematic review and meta-analysis. Auris Nasus Larynx. (2025) 52(1):28–34. 10.1016/j.anl.2024.12.004 39787949

[10] Wang EW Gardner PA Fraser S Stefko ST Fernandez-Miranda JC Snyderman CH. Reduced tearing with stable quality of life after vidian neurectomy: a prospective controlled trial. Laryngoscope. (2021) 131(7):1487–91. 10.1002/lary.29287 33247625

[11] Choi RY Coyner AS Kalpathy-Cramer J Chiang MF Campbell JP. Introduction to machine learning, neural networks, and deep learning. Transl Vis Sci Technol. (2020) 9:2–14. 10.1167/tvst.9.2.14 PMC7347027 32704420

[12] Zuo D Yang L Jin Y Qi H Liu Y Ren L. Machine learning-based models for the prediction of breast cancer recurrence risk. BMC Med Inform Decis Mak. (2023) 23:1–276. 10.1186/s12911-023-02377-z 38031071 PMC10688055

[13] Gu Y Su S Wang X Mao J Ni X Li A Comparative study of XGBoost and logistic regression for predicting sarcopenia in postsurgical gastric cancer patients. Sci Rep. (2025) 15:1–12808. 10.1038/s41598-025-98075-z 40229548 PMC11997166

[14] Sun S Chen A Shi L Wan Y. Two types of vidian neurectomy show efficacy in treating allergic rhinitis and vasomotor rhinitis. Sci Rep. (2024) 14(1):27303. 10.1038/s41598-024-78116-9 39516526 PMC11549493

[15] Brożek JL Bousquet J Agache I Agarwal A Bachert C Bosnic-Anticevich S Allergic rhinitis and its impact on asthma (ARIA) guidelines-2016 revision. J Allergy Clin Immunol. (2017) 140(4):950–8. 10.1016/j.jaci.2017.03.050 28602936

[16] Tepeš I Košak Soklič T Urbančič J. The agreement of the endoscopic modified Lund-kennedy scoring in a clinical research group: an observational study. Eur Ann Otorhinolaryngol Head Neck Dis. (2022) 139(4):185–8. 10.1016/j.anorl.2021.08.014 34654664

[17] Brescia G Sfriso P Marioni G. Role of blood inflammatory cells in chronic rhinosinusitis with nasal polyps. Acta Otolaryngol. (2019) 139(1):48–51. 10.1080/00016489.2018.1538567 30686139

[18] Sheinenzon A Shehadeh M Michelis R Shaoul E Ronen O. Serum albumin levels and inflammation. Int J Biol Macromol. (2021) 184:857–62. 10.1016/j.ijbiomac.2021.06.140 34181998

[19] Iwasaki N Poposki JA Oka A Kidoguchi M Klingler AI Suh LA Single cell RNA sequencing of human eosinophils from nasal polyps reveals eosinophil heterogeneity in chronic rhinosinusitis tissue. J Allergy Clin Immunol. (2024) 154(4):952–64. 10.1016/j.jaci.2024.05.014 38797240 PMC11456383

[20] Shin SH Ye MK Park J Geum SY. Immunopathologic role of eosinophils in eosinophilic chronic rhinosinusitis. Int J Mol Sci. (2022) 23:21. 10.3390/ijms232113313 PMC9658199 36362100

[21] Ramadani F Bowen H Upton N Hobson PS Chan YC Chen JB Ontogeny of human IgE-expressing B cells and plasma cells. Allergy. (2017) 72(1):66–76. 10.1111/all.12911 27061189 PMC5107308

[22] Qiu C Zhong L Huang C Long J Ye X Wu J Cell-bound IgE and plasma IgE as a combined clinical diagnostic indicator for allergic patients. Sci Rep. (2020) 10:1–4700. 10.1038/s41598-020-61455-8 32170187 PMC7069990

[23] Wang W Xu Y Wang L Zhu Z Aodeng S Chen H Single-cell profiling identifies mechanisms of inflammatory heterogeneity in chronic rhinosinusitis. Nat Immunol. (2022) 23(10):1484–94. 10.1038/s41590-022-01312-0 36138182

[24] Plath M Sand M Cavaliere C Plinkert PK Baumann I Zaoui K. Normative data for interpreting the SNOT-22. Acta Otorhinolaryngol Ital. (2023) 43(6):390–9. 10.14639/0392-100x-n2279 37814974 PMC10773542

[25] Liu M Liu J Weitzel EK Chen PG. The predictive utility of the 22-item sino-nasal outcome test (SNOT-22): a scoping review. Int Forum Allergy Rhinol. (2022) 12(1):83–102. 10.1002/alr.22888 34585521

[26] Kim DH Jang DW Hwang SH. Efficacy of additional (selective) vidian neurectomy in treating chronic rhinosinusitis with nasal polyp: a systematic review and meta-analysis. ORL J Otorhinolaryngol Relat Spec. (2025) 87(1):57–68. 10.1159/000546116 40300573 PMC12158399

