# Supplementary figures and images for: Development and validation of a nomogram for predicting the efficacy of vidian neurectomy in the treatment of chronic rhinosinusitis with nasal polyps combined with allergic rhinitis
Source: Front Surg. 2025 Nov 18;12:1682674. doi: 10.3389/fsurg.2025.1682674 (PMC12746656; doi:10.3389/fsurg.2025.1682674)

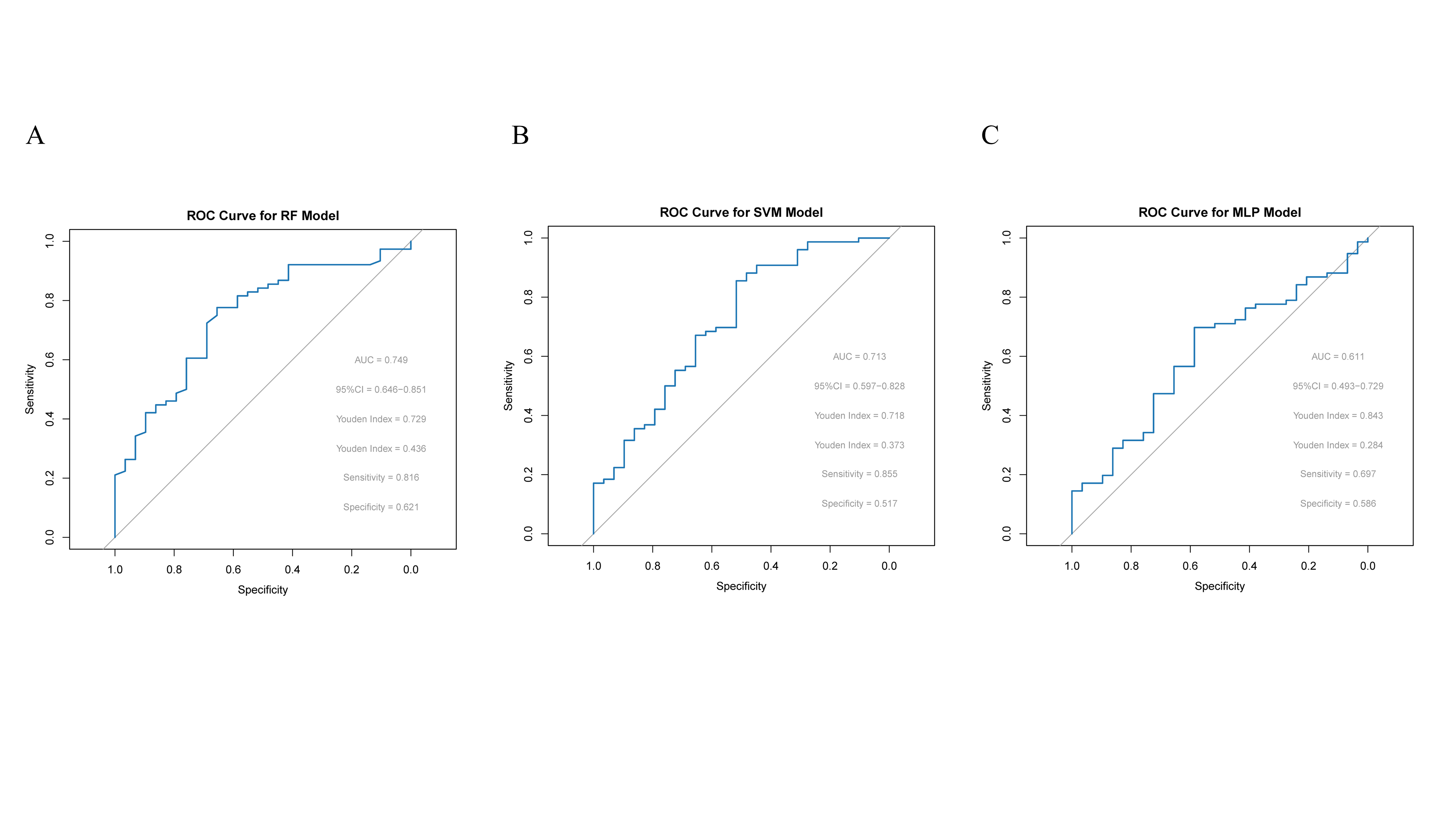

Supplement: Supplementary Figure S1 — (A) ROC curve for RF model (B) ROC curve for SVM model (C) ROC curve for MLP model. [file Image1.tif]
