# Supplementary material for: Development and validation of a nomogram for predicting the efficacy of vidian neurectomy in the treatment of chronic rhinosinusitis with nasal polyps combined with allergic rhinitis
Source: Front Surg. 2025 Nov 18;12:1682674. doi: 10.3389/fsurg.2025.1682674 (PMC12746656; doi:10.3389/fsurg.2025.1682674)
Supplement: Supplementary file 2 [file Table1.docx]

| **Supplementary Table. 1 Comparison of Classification Performance Metrics of Machine Learning Models** | |
| --- | --- |
| **Term** | **Value** |
| **XGBoost** |  |
| **Accuracy** | 0.800 |
| **Recall** | 0.922 |
| **Precision** | 0.826 |
| **F1 Score** | 0.871 |
| **RF** |  |
| **Accuracy** | 0.724 |
| **Recall** | 0.974 |
| **Precision** | 0.733 |
| **F1 Score** | 0.836 |
| **SVM** |  |
| **Accuracy** | 0.752 |
| **Recall** | 0.987 |
| **Precision** | 0.750 |
| **F1 Score** | 0.852 |
| **MLP** |  |
| **Accuracy** | 0.686 |
| **Recall** | 0.868 |
| **Precision** | 0.742 |
| **F1 Score** | 0.800 |
